# Using qualitative comparative analysis to uncover multiple pathways to program sustainment: implications for community-based youth substance misuse prevention

**DOI:** 10.1186/s43058-022-00303-4

**Published:** 2022-05-26

**Authors:** Brittany Rhoades Cooper, Laura G. Hill, Louise Parker, Garrett J. Jenkins, Gitanjali Shrestha, Angie Funaiole

**Affiliations:** 1 Department of Human Development, Washington State University, Pullman, WA USA; 2 IMPACT Research Lab, Pullman, WA USA; 3 Washington State Department of Health, Olympia, WA USA

**Keywords:** Evidence-based prevention, Sustainment, Sustainability, Qualitative comparative analysis

## Abstract

**Background:**

In order to achieve wide-scale impact in community settings, programs must be sustained. Theory and empirical evidence suggest that intervention characteristics, organizational context, capacity for program implementation, and processes related to implementation are associated with continued program delivery. However, few studies examine how combinations of these factors work together in different settings to influence program sustainment.

**Methods:**

Using scales specified in the Program Sustainability Assessment Tool (PSAT), the current cross-sectional study aims to identify the necessary and sufficient conditions for the sustainment of the Strengthening Families Program for Parents and Youth 10-14 (SFP 10-14). Staff (*n* = 59) at SFP 10-14 implementation sites across Washington State completed an online survey reporting on their current level of SFP 10-14 sustainment. They also completed PSAT, with eight scales designed to assess conditions that consistently produce sustainment. Data were analyzed using qualitative comparative analysis.

**Results:**

Environmental support was the only necessary condition for sustainment success. Four solutions sufficient to achieve sustainment were also identified. These included the combined presence of (1) environmental support, organizational capacity, and funding stability; (2) environmental support, organizational capacity, communication, and program evaluation, in the absence of strategic planning; (3) environmental support, organizational capacity, program evaluation, and partnerships, in the absence of strategic planning; and (4) environmental support, communication, partnerships, and funding stability, in the absence of program evaluation.

**Conclusions:**

Environmental support in combination with organizational capacity appeared to most consistently produce sustainment of SFP 10-14 programs in Washington State. Program providers will benefit from a focusing on enhancing those conditions to increase program sustainment.

Contributions to the literature

- This study builds on the growing body of empirical studies that use a validated quantitative instrument and conceptual framework (i.e., the Program Sustainability Assessment Tool) to understand antecedents of program sustainment.
- This study uses Qualitative Comparative Analysis to improve understanding of which combinations must be present and which are sufficient for the program sustainment of a community-based youth substance misuse prevention program.
- Results from this study can begin to inform the development and testing of evidence-based sustainment strategies and support systems that acknowledge and allow for the various needs of diverse programs working in diverse settings.

## Background

Nearly all the most common causes of morbidity and mortality in the USA are preventable [1]. As a result, the federal government has called for increased emphasis on the development, evaluation, and large-scale dissemination of evidence-based prevention programs aimed at reducing the risks for important social problems like adolescent substance use and abuse [2]. Adolescence is typically described as the developmental period between childhood and adulthood and can include youth age 10 through early adulthood (i.e., age 24) [3]. Early preventive interventions are especially relevant during this time period as more than 29% of 12th graders report using alcohol in the past 30 days and over one third reported using marijuana in the past year [4]. Therefore, early adolescence is an important transition point where intervention can most effectively prevent later substance misuse. The negative effects of adolescent substance use and misuse are well-documented and include both adverse health consequences (e.g., unintentional injury) [5] and social (e.g., poor relationships) and economic (e.g., lost productivity) costs for society [6]. The prevalent use of substances during adolescence and associated negative consequences has spurred much research on effective educational and behavioral prevention efforts. Fortunately, significant progress has been made; today several programs have demonstrated efficacy in the reduction of youth risk behaviors and have been widely promoted through the allocation of federal, state, and philanthropic dollars [7, 8].

### Strengthening Families for Parents and Youth 10-14

Strengthening Families Program for Parents and Youth 10-14 (SFP 10-14), an internationally recognized evidence-based family skills training program for youth ages 10–14 and their caregivers, is one of the leading adolescent substance misuse abuse prevention programs in the country. In a systematic review of 56 clinical trials of substance use prevention programs, Foxcraft and colleagues [9] found that SFP 10-14 was the only family-based substance use prevention program deemed to have long-term efficacy. The program has demonstrated consistent positive impacts on youth behavior problems, delinquency, and alcohol and drug abuse in clinical trials [10–12]. For example, Spoth et al. [11] found that youth participants in SFP 10-14 were about half as likely as those who did not receive SFP 10-14 to report ever using alcohol or ever being drunk 2 years following the end of the program.

In addition to having a strong evidence base, SFP 10-14 is also one of the most widely disseminated evidence-based programs in the country and across Washington State. For over 15 years, Washington State University and community partners have trained SFP 10-14 facilitators who have implemented nearly 650 programs and reached nearly 11,000 caregivers and youth. The program is typically delivered by community-based organizations (e.g., youth- and family-serving agencies) often in partnership with a local school or school district that can serve as a source for recruiting families to participate. These organizations often receive grant funds to coordinate and implement the program, including providing hourly pay for two to four certified facilitators to coordinate and deliver the program to parents and youth in 2 h, once-a-week sessions for 7 weeks. The location for the sessions is often at schools, community centers, or houses of faith in the evenings. Most organizations choose to implement a single 7-week program to 10–12 families at a time.

Although wide dissemination is important, programs like SFP 10-14 are unlikely to have a measurable impact on the critical social problems of today if they are not sustained beyond the initial start-up grant dollars allocated to promote their adoption [13]. Sustainability, or program sustainment, is defined as the “continued use of the program components and activities for the continued achievement of desirable program and population outcomes” [14]. Because most community-based prevention funding is distributed through time-limited grants intended to “seed” effective prevention efforts, the long-term enduring success and public health impact of substance use prevention efforts are dependent upon programs’ abilities to sustain their efforts beyond initial seed grant funding.

### Factors associated with program sustainment

As was documented in a systematic review of 125 empirical studies of health-focused interventions, most sustainability research focuses on categorizing factors presumably associated with program continuation—known as program sustainment [15]. From this review and a recent conceptual model proposed by Shelton, Cooper, & Wiltsey Stirman [16], we know that the characteristics of the interventions (e.g., the fit with the implementing organization, ability to be modified, perceived effectiveness, and ability to be implemented with fidelity), organizational context (e.g., climate, leadership, infrastructure), capacity for program implementation (e.g., funding, staff, stakeholder involvement), and processes related to implementation (e.g., relationship building, evaluation, planning) play an important role. However, this knowledge is greatly limited because most studies included small, homogeneous samples, and their conclusions were mostly based on qualitative retrospective assessments of individuals’ beliefs about what led to sustainment success or failure, rather than based on statistical tests of their association [17–22].

Those that were more quantitative in nature rarely utilized validated instruments to measure predictors [23], and despite acknowledgement of the likely interaction of these factors and multiple pathways to sustainment, few existing studies model this complexity [24, 25]. There are some exceptions, however [26, 27]. For example, Welsh and colleagues examined aspects of collaboration and organizational functioning as predictors in a longitudinal study of the financial sustainability of 14 community coalitions responsible for implementing youth-focused and the family-focused SFP 10-14 substance use prevention programs across two states. They found that the amount of program implementation funds raised over 5 years was predicted by earlier and concurrent team functioning and sustainability planning. However, by year 8, predictors varied across states with a strong positive association between team functioning and total funds raised in one state, and a negative association in the other state. The authors concluded that program sustainment was largely a “local process” resulting from different relationships in different communities with different resources and infrastructure [27].

### Conceptual framework

The present study used the Capacity for Sustainability Framework and its resultant Program Sustainability Assessment Tool (PSAT) to guide data collection, analysis, and interpretation [28, 29]. Luke and colleagues define sustainability or sustainment as “a set of organizational and contextual factors that build the capacity for maintaining a public health program over time” (pg. 2). They conducted factor analysis with a sample of 592 participants from 252 state and community public health programs that yielded eight PSAT domains—environmental support, funding stability, partnerships, organizational capacity, program evaluation, program adaptation, communications, and strategic planning—with good discriminant and construct validity [28]. Since its inception, the PSAT has been employed in several sustainability studies, though the studies have been primarily descriptive, seldom examining the predictive strength between the PSAT domains and program sustainment [30, 31]. One exception is the study by Hunter and colleagues [32], based on reports from 169 staff within 78 organizations, which demonstrated the ability of six of the PSAT scales to predict the sustainment of an evidence-based youth substance use treatment program. They found that higher levels of communication, funding stability, partnerships, environmental support, organizational capacity, and strategic planning were related to the continuation of the Adolescent-Community Reinforcement Approach program after the discontinuation of initial implementation support.

### Current study

Using the scales specified in the PSAT and the aforementioned definition of sustainment, the current study aims to enhance our understanding of explanatory factors associated with sustainment by identifying the necessary (i.e., the condition is nearly always present when sustainment occurs) and sufficient (i.e., sustainment is nearly always evident when the condition occurs) conditions for the continued delivery of SFP 10-14 among a sample of Washington State sites that have all successfully implemented SFP 10-14 in the past, but that vary in their current level of successful sustainment. We analyzed survey data collected from staff at SFP 10-14 implementation sites using crisp-set Qualitative Comparative Analysis (QCA), which assesses the quantitative associations between configurations of conditions and the presence or absence of an outcome [33].

## Methods

### Study participants

We identified potential study participations through two existing databases—the Washington State University SFP 10-14 evaluation database and the Washington State Division of Behavioral Health and Recovery prevention services database. Both databases included names and contact information for SFP 10-14 coordinators and facilitators that were successfully implementing or in the past had successfully implemented at least one instance of the 7-week SFP 10-14 program in Washington State. Recruitment emails were sent by the first author to all contacts that provided email addresses (*N* = 119). Participants were eligible to complete the cross-sectional survey if they considered themselves coordinators, facilitators, or other staff associated with at least one successful delivery of the 7-week SFP 10-14 program and responded to the survey within the data collection period. Of the 119 contacted, 59 (49.6%) staff from SFP 10-14 sites across Washington State completed an online survey between January and February 2015 assessing factors associated with the sustainment as well as level of current and future program sustainment. Eighty-nine percent of respondents were female: 77% were White (non-Hispanic) and 16% were Hispanic/Latino, 79% had earned a Bachelor’s degree or higher, and 48% had been a family educator for 6 years or more. In addition, 79% lived in the same community in which they implemented SFP 10-14, 87% were financially or otherwise compensated for their SFP 10-14 work, 91% reported that SFP 10-14-related duties were not their primary job, and 32% reported delivering the program to special/targeted populations. The characteristics of this study sample closely resemble other SFP 10-14 facilitator samples [34, 35]. Sixty-seven percent of participants reported implementing SFP 10-14 in English and 33% in both English and Spanish; 65% reported implementing the program in a school. Participants identified their own roles in SFP 10-14 delivery as 27% facilitator, 32% co-facilitator, 33% site coordinator, 15% program coordinator (multiple sites), and 27% other. Due to the varying duties and roles implementers often assume in natural contexts, participants could select all applicable descriptions. SFP 10-14 staff were associated with a wide variety of organizations, including school districts, county health departments, and substance misuse prevention coalitions.

### Measures

#### Sustainment outcome

In line with the Capacity for Sustainability Framework and Pluye et al.’s [36] conceptualization of sustainability levels, we developed one item that asked respondents: Based on the descriptions provided, what level best describes your experience with SFP 10-14 at your site? All levels indicate successful initial implementation (i.e., delivery) of SFP 10-14, but response options distinguish their degree of successful sustainment from absent to routinized: Level 1=We have delivered the SFP 10-14 program in the past, but we are not currently delivering SFP 10-14 programs (Absent); Level 2=We have delivered the SFP 10-14 program in the past; currently, we are providing family/parenting classes to parents of adolescents, but we are not delivering the SFP 10-14 program model specifically (Precarious); Level 3=We have delivered at least one SFP 10-14 program in the last year, but the staff and resources needed to successfully deliver the program have not been well supported by our organization; it is a struggle to consistently offer SFP 10-14 (Weak); Level 4=We have consistently delivered SFP 10-14 in the past and plan to continue; and the staff and resources needed to successfully deliver SFP 10-14 are well supported and integrated into the normal operations of our organization (Routinized).

#### Sustainment conditions

Similar to past studies using the PSAT [30, 37], information on the conditions related to program sustainment was collected via individual self-report on the eight PSAT scales: environmental support, funding stability, partnerships, organizational capacity, program evaluation, program adaptation, communications, and strategic planning. A description, example items, and Cronbach’s alpha for each scale are included in Table 1. Each scale contained five items for a total of 40 items. For each item, participants were presented with a statement and asked to select the number on a 7-point scale (1=to little or no extent, 7=to a very great extent) that best indicated the extent to which their program has or does that particular thing (e.g., champions exist who strongly support our program). Participants were also given the option to answer “not able to answer”—these responses were coded as missing and not included in the calculation of the mean scale scores.

**Table 1:** Program sustainability assessment tool scales and correlations with sustainment level

Scale	Description	Example item	Cronbach’s alpha	M (SD)	*r*
Environmental Support	Supportive internal and external climate for your program	We have strong champions with the ability to garner resources.	0.92	4.84 (1.59)	.69***
Funding Stability	Consistent financial base for your program	My program exists in a supportive state economic climate.	0.92	4.01 (1.75)	.64***
Partnerships	Connections between your program and its stakeholders	Diverse community organizations are invested in the success of our program.	0.94	4.26 (1.71)	.71***
Organizational Capacity	Internal support and resources needed to effectively manage your program	Organizational systems are in place to support our program needs.	0.92	5.21 (1.48)	.59***
Program Evaluation	Assessing your program to inform planning and document results	We have the capacity for quality program evaluation.	0.90	5.36 (1.31)	.42***
Program Adaptation	Using empirical and experiential information to adapt the program to fit changing contexts and conditions	We adapt strategies as needed.	0.91	5.01 (1.47)	.25
Communication	Strategic communication with stakeholders and the public about your program	We communicate the need for the program to the public.	0.96	4.58 (1.62)	.62***
Strategic Planning	Processes that guide your program’s direction, goals, and strategies	We plan for future resource needs.	0.89	4.45 (1.75)	.44**

*N* = 23–40

**p < .01

***p < .001

### Analytic approach: a Qualitative Comparative Analysis (QCA)

QCA is a case-oriented approach that uses Boolean algebra to examine relationships among antecedents to an outcome. QCA provides an advantage over parametric statistics by allowing cross-case comparisons showing how the presence or absence of a condition (i.e., explanatory factor) influences the observation of an outcome. Additionally, QCA is not burdened by the same sample size constraints that parametric statistics must navigate [38]. QCA can examine complex combinations of explanatory factors (or pathways) across differing contexts and produce externally valid results even among a smaller sample [39–41]. In the present study, QCA gives us the opportunity to examine how combinations of conditions are related to SFP 10-14 sustainment differently across sites—therefore illuminating different possible pathways to sustainment (i.e., equifinality). It also allows us to explore whether certain factors are only relevant to sustainment success when in combination (i.e., casual complexity). Other variable-centered methods (i.e., regression) would not be able to detect these complex relationships.

There are various approaches to QCA (e.g., crisp-set, fuzzy-set); however, as noted by several implementation researchers, crisp-set QCA can improve interpretation and provide clearer practice implications [33, 40]. For this reason and because our interest is in differences in “kind” as opposed to differences in “degree” of the included conditions and how their combinations produce sustainment, we chose crisp-set over fuzzy-set QCA (Rohlfing, 2020). Also, there is some precedence for using crisp-set QCA with PSAT scores and we aimed to compare our findings with this previous work [42].

#### Calibration

Crisp-set QCA requires conditions and outcomes to be dichotomized (1 = present, 0 = absent) and it requires complete data for each case; only cases with complete data for each analysis will be retained. See below for details. For this study, all conditions measured by the PSAT scales were dichotomized via mean-split, with average or greater levels indicating the presence of a condition. The program sustainment outcome was dichotomized such that any SFP 10-14 implementation site indicating their SFP 10-14 implementation was routinized (Level 4) was considered sustained and denoted as present. The remaining levels (1–3) were coded as absent. Details are presented in the results section.

#### Truth tables

After calibration of the data, truth tables were created. Each row in the truth table represents the configuration of conditions and outcome present for a specific respondent (i.e., case). Several cases may share the same configuration of conditions. For each configuration that was represented by at least one case, a raw consistency value was obtained. This value reflects the percentage of cases sharing that configuration of conditions that also had achieved program sustainment. For instance, in our analyses, one configuration was common to six separate cases. Five of these cases achieved program sustainment, while one did not. As a result, the consistency value is noted as 0.83 (five out of six). The software used, fs/QCA, defaults to an inclusionary consistency cutoff value of 0.8, which was used in the present study.

#### Necessity analysis

Following the construction of the truth table, configurations which demonstrated less than a 0.8 consistency in achieving the outcome of sustainment were removed. A necessity analysis was then undertaken in which all redundant conditions were also removed. These “non-difference-making factors” [43] are those which do not demonstrate a highly consistent presence across all conditional configurations when the outcome is observed. Though a consistency value of 0.8 is accepted as a suitable benchmark for initial inclusion in QCA analyses, a significantly greater value should be observed when making claims about the necessity of a condition [44]. Though no specific guideline has been agreed upon, this study used a threshold of 0.9. Therefore, when a condition is referred to as necessary it indicates that the condition preceded the outcome a minimum of nine out of ten times.

#### Sufficiency analysis

Any condition, or combination of conditions, may be considered sufficient when it includes the necessary condition(s) and also demonstrates the outcome of program sustainment. Sufficiency analyses may generate three sets of solutions: complex, intermediate, and parsimonious. The debate has transpired over the last decade about which solution set to report, with significant expert disagreement [43, 45, 46]. Our group agrees with the position of Ragin and Sonnett [46] that the intermediate solution provides an advantageous balance of parsimony and complexity that is at once concise, yet flexible enough to account for diverse contexts and therefore we will report the intermediate solution.

#### Model fit

Determining the validity of the solutions generated by QCA was accomplished by examining two indices of model fit: solution coverage and solution consistency. Coverage may be conceptualized much the same as variance explained in parametric statistics. Values range from 0 to 1, where higher values demonstrate equivalently greater empirical relevance [47]. No minimum values are required. Consistency assesses the frequency with which a combination is sufficient to achieve sustainment in fact achieves the outcome. A value of 0.8 is recommended as a minimum guideline for claims of causality [48] and therefore is used in this study.

## Results

### Calibration

According to Kahwati & Kane [49], researchers should use existing theory/research, empirical evidence, and practical considerations when deciding which and how many conditions to include in QCA. They also recommend that most QCA models should not exceed six total conditions. Because the PSAT measure included eight scales, we aimed to reduce our number of conditions prior to analysis. Because the PSAT was developed for use with disease prevention public health programs, we began by examining the face validity of each scale for the present sample. All scales except the adaptation scale were deemed to have face validity for the SFP 10-14 sample. SFP 10-14 is a highly manualized program and program facilitators are strongly encouraged to maintain high fidelity, with few if any adaptations in order to assure high-quality program delivery and outcomes. Therefore, we would not expect adaptation to be related to SFP 10-14 sustainment. We also ran correlations between the ordinal version of the program sustainment outcome (absent, precarious, weak, or routinized) and the scales of the PSAT. Results showed that all PSAT scales, except program adaptation, were significantly positively correlated with program sustainment (see Table 1). Due to a lack of empirical support for the relevance of this scale, the lack of face validity for the present sample, and the demands an extra condition creates in QCA (e.g., “limited diversity” [44]), the adaptation scale was excluded from the following analyses. As described above, for the purposes of crisp-set QCA analysis, all conditions and the outcome must be dichotomized. Frequencies for the mean-split versions of the PSAT scales—i.e., conditions—and the dichotomous version of the program sustainment outcomes are displayed in Table 2. QCA requires complete data and therefore only cases with complete data are represented in the results presented below. In eleven cases (18.3%), individuals did not respond to the item that assessed the sustainment outcome and so could not be included in the analysis. Across the PSAT scales between 16 and 21 individuals did not respond and so were similarly excluded from analysis, providing a final analytic sample size of 32 cases. This analytic sample did not significantly differ from those with missing data on a variety of individual characteristics including: gender, race/ethnicity, level of education, whether they lived in the community where the program was delivered, whether they were compensated for their SFP 10-14 work, how much experience they had as a parent education/group facilitator, or whether their work with SFP 10-14 was their primary job. They also did not significantly differ on a variety of program characteristics including the version of the program implemented (English vs. Spanish), whether the program was targeted at higher-risk populations, or where the program was being delivered (e.g., school, health center, house of faith). The truth table for these cases is displayed in Table 3. In all, 14 (43.7%) of the 32 cases successfully achieved sustainment.

**Table 2:** Frequencies and percentages for dichotomized PSAT conditions and program sustainment outcome

QCA conditions and outcome	Number of cases (%) absent	Number of cases (%) present
PSAT conditions		
Environmental support	17 (38.6%)	27 (61.4%)
Funding stability	21 (50.0%)	21 (50.0%)
Partnerships	19 (46.3%)	22 (53.7%)
Organizational capacity	19 (43.2%)	25 (56.8%)
Evaluation	21 (47.7%)	23 (52.3%)
Adaptations	21 (48.8%)	22 (51.2%)
Communication	19 (44.2%)	24 (55.8%)
Strategic planning	20 (51.3%)	19 (48.7%)
Program sustainment outcome		
Routinized	21 (42.9%)	28 (57.1%)

*N* = 39–49

**Table 3:** Truth table

Number of cases	PSAT conditions							Outcome	Consistency score
	Support	Fund	Partner	Org Cap	Eval	Comm	Plan	Routinized sustainment	
1	1	0	0	1	1	1	0	1	1.0
1	1	0	1	1	1	0	0	1	1.0
1	1	1	0	1	0	0	0	1	1.0
1	1	1	0	1	0	1	0	1	1.0
1	1	1	0	1	1	1	0	1	1.0
1	1	1	1	0	0	1	0	1	1.0
1	1	1	1	0	0	1	1	1	1.0
1	1	1	1	1	0	1	1	1	1.0
6	1	1	1	1	1	1	1	1	.83
3	1	0	1	1	1	1	1	0	.67
2	1	1	1	0	1	1	1	0	.50
11	0	0	0	0	0	0	0	0	.09
1	0	0	0	1	0	0	0	0	0.0
1	0	1	0	0	0	0	0	0	0.0

*N* = 32; *Support* environmental support, *Fund* funding stability, *Partner* partnerships, *Org Cap* organizational capacity, *Eval* program evaluation, *Comm* communication, *Plan* strategic planning

### Necessity analysis

We specified a consistency value of 0.9 or greater to indicate conditional necessity. Environmental support was the only condition that met this criterion, with 94% of the cases with average or above environmental support also reporting routinized SFP 10-14 sustainment (see Table 4).

**Table 4:** Results of necessity and sufficiency analyses

PSAT Condition	Necessity analysis		
	Consistency	Coverage	
Environmental support	.94	.84	
Funding stability	.71	.80	
Partnerships	.71	.80	
Organizational capacity	.76	.81	
Program evaluation	.65	.79	
Communication	.82	.82	
Strategic planning	.59	.77	
PSAT conditions	Sufficiency analysis: intermediate solution		
	Consistency	Raw coverage	Unique coverage
1. Support * Org Cap * Fund	.90	.53	.41
2. Support * Org Cap * Comm * Eval * ~Plan	1	.12	.06
3. Support * Org Cap * Eval * Partner * ~Plan	1	.06	.06
4. Support * Comm * Partner * Fund * ~Eval	1	.18	.12
Total		.76	.93

*N* = 32; *Support* environmental support, *Fund* funding stability, *Partner* partnerships, *Org Cap* organizational capacity, *Eval* program evaluation, *Comm* communication, *Plan* strategic planning

~Denotes the absence of that condition

### Sufficiency analysis and model fit

Four solutions sufficient to achieve sustainment were identified in the intermediate solution, each with some unique coverage, or variance explained (see Table 4). By far, the most substantively significant was Solution 1, which had the greatest amounts of raw and unique coverage, as well as the fewest conditional requirements for achievement of program sustainment. Solution 1 showed that the combined presence of environmental support, organizational capacity, and funding stability was sufficient to achieve program sustainment in 53% of total cases. This combination of conditions demonstrated high causal consistency, with 90% of cases in which this pattern was present achieving program sustainment. Solution 2 showed that the combined presence of environmental support, organizational capacity, communication, and program evaluation (in the absence of strategic planning) was sufficient to achieve program sustainment in 12% of total cases. This combination of conditions demonstrated perfect causal consistency, with 100% of cases in which this pattern was present achieving program sustainment. Solution 3 showed that the combined presence of environmental support, organizational capacity, program evaluation, and partnerships (in the absence of strategic planning) was sufficient to achieve program sustainment in 6% of total cases. This combination of conditions demonstrated perfect causal consistency, with 100% of cases in which this pattern was present achieving program sustainment. Solution 4 showed that the combined presence of environmental support, communication, partnerships, and funding stability (in the absence of program evaluation) was sufficient to achieve program sustainment in 18% of total cases. This combination also demonstrated perfect causal consistency.

In line with suggestions by Schneider and Wagemann [44], we ran additional analyses to determine what conditions consistently produced sustainment failure as well, which had a necessity consistency of 0.2, well below our 0.9 threshold for necessity claims. Further, both the parsimonious and intermediate solutions for the negation analyses report the only condition sufficient for sustainment failure is the absence of environmental support. That the absence of environmental support should consistently produce sustainment failure further fortifies our finding that the presence of it is necessary to sustain SFP 10-14.

## Discussion

In this study, we explored the factors associated with the sustainment of a community-based, family-focused prevention program. Environmental support—the presence of a supportive internal and external climate for the program—stands out among the eight PSAT domains included in this study because it is the only one that proved necessary for program sustainment. Our findings are consistent with previous research using the PSAT showing higher levels of environmental support are reported by programs with greater sustainment success [31, 32, 42]. Most of this existing research, however, is qualitative in nature, and those who use quantitative techniques focus on measures of association, or covariation, between variables. QCA differs in its base objectives: to determine the conditions, or combinations of conditions, which are necessary and/or sufficient to realize an outcome [45]. For example, in our study, we found that when simply examining correlations between PSAT domains and sustainment success, nearly every domain (apart from program adaptations) predicted sustainment, but when we examined the effect of different combinations of conditions using QCA analyses, we found a more nuanced story. While the partnership scale was the condition most strongly correlated with the sustainment outcome (see Table 1), under QCA analyses its importance is considerably less than other conditions. Partnerships accompanied sustainment in 71% of cases, less frequently than did environmental support (94%), organizational capacity (76%), and communication (82%), and only contributed to attainment of the sustainment outcome in 24% of cases. Both environmental support (76%) and organizational capacity (71%) far surpassed this rate of contribution. As Fleiszer et al. [17] note, “sustainability is likely contingent on the interplay of multiple factors at different levels of analysis, points in time and settings, and that these interactive effects make it difficult to establish the relative importance of individual factors”. Using QCA, the present study found that (a) there are multiple possible pathways to sustainment (i.e., with varying combinations of PSAT domains) and (b) some PSAT domains appear to have more relevance than others.

Specifically, we found that environmental support, in combination with other domains, consistently produced sustainment in all four solutions identified in this study. And in three out of the four solutions, the combination of environmental support and organizational capacity (i.e., internal support and resources needed to effectively manage your program) was present. These findings align with existing implementation and sustainment frameworks. The Exploration, Preparation, Implementation, and Sustainment (EPIS) model of implementation [50] and the Integrated Sustainability Framework [16] have recognized inner (e.g., inter-organizational support and champions, adequate staff) and outer (e.g., socio-political context and funding environment) contextual factors predictive of successful initial and continued program implementation. Empirical research also shows that the presence of internal and external champions, supporters, and advocates influences program sustainment [26, 51–53] and that organizational capacity, such as having adequate resources and staff to deliver the program [19, 24, 31, 53] is integral to a program’s success and continued delivery. Further, a recent study by King and colleagues [42] who conducted a similar QCA analysis using dichotomized versions of PSAT domains as conditions to examine necessary and sufficient pathways to the sustainment of an alcohol screening and brief intervention found that strong environmental support often coincided with strong organizational capacity.

The role of environmental support in combination with organizational capacity may be especially important for the sustainment of community-based, family-focused programs like SFP 10-14. Scheirer et al. [54] posit an intervention typology based on who and what is required for continued implementation—which hypothesizes what factors are most salient for sustainment success of each type. They describe six intervention types—one of which includes interventions like SFP 10-14 that require coordination of multiple types of staff implemented by organizations in community settings. As opposed to other types (e.g., interventions implemented by individual providers), they hypothesize that organizational leadership, support, and capacity are critical to both initial and ongoing implementation. This is certainly in line with findings from the present study. Strong support and capacity for SFP 10-14 within the organization and in the community are particularly important to foster a sense of program ownership which, in turn, may contribute to the integration of the program into the organization and the community at large.

Funding is often identified as the major barrier to program sustainment [20, 31, 55]. Interestingly, our study found that funding stability—having a consistent financial base for your program—was not a necessary condition for sustainment and was only present in two of the four sufficiency solutions. This finding appears to be in line with a study by Tabak and colleagues [31] that found both sites with high and low sustainment capacity reporting limited funding stability. In their study on the sustainability potential of an alcohol screening and brief intervention program within three primary care systems, King and colleagues [42] found that all non-sustaining primary care systems had low levels of funding stability. However, they also found evidence of weak funding stability in one site exhibiting sustainment success. The authors hypothesized that funding challenges could potentially be overcome by strengthening factors in other sustainability domains such as increasing commitment to the program and by adapting programs to ensure efficient integration into the current practices and workflows. Our study provides additional empirical evidence that funding stability is not a necessary condition for program sustainment and that exclusive emphasis on funding stability can overshadow the compensatory strength of other domains. Programs with greater than average levels of funding stability in our study achieved program sustainment in only 71% of the cases. Thus, the results support the assertion that strengthening other sustainability domains may help contribute to program sustainment even in the face of weak financial stability [27, 42].

Another surprising finding from the current study was that the absence of strategic planning was associated with program sustainment. In the PSAT, strategic planning is measured by five items: (1) the program plans for future resource needs, (2) the program has a long-term financial plan, (3) the program has a sustainability plan, (4) the program’s goals are understood by all stakeholders, and (5) the program clearly outlines roles and responsibilities for all stakeholders. Items 1, 2, and 3 are particularly suggestive of sustainment success and also had the highest factor loadings in Luke et al.’s [28] initial psychometric evaluation of the strategic planning scale. In both solutions where the absence of strategic planning was a condition associated with sustainment, however, program evaluation, environmental support, and organizational capacity were present. These domains contain items that could make strategic planning redundant. For instance, the program evaluation domain contains an item assessing the ability to demonstrate success to funders. Environmental support contains an item regarding resource mobilization. Organizational capacity contains an item about effective resource management. Thus, it is also possible that the presence of this combination of domains compensates for the absence of strategic planning.

Program evaluation has been consistently recognized in the literature as a key ingredient for program sustainment [18, 21, 56]. In a study conducted by King and colleagues [42], program evaluation was the only domain which was rated consistently strong for all sustaining sites and consistently weak for all non-sustaining sites. Similarly, Tabak and colleagues [31] found that high-capacity sites integrated program evaluation into their implementation and sustainability, while low-capacity sites reported limited evaluation capacity. The results from the current study are mixed regarding program evaluation. Consistent with the literature, program evaluation consistently produced sustainment in combination with other domains in two of the four sufficiency solutions. However, in another of the sufficiency solutions, program evaluation was required to be absent, while environmental support, communication, partnership, and funding stability was required to be present. The results of program evaluation are usually used to garner support for the program, create or strengthen partnerships, provide information about the program, create interest in the program, and show progress to the funder. In the absence of program evaluation, the presence of the other four domains may have provided compensatory strength for program sustainment [30].

### Strengths and limitations

Our study contributes to the growing literature which uses QCA to understand and quantify complex implementation constructs [33, 41, 42, 57, 58]. Hill and colleagues [33] used QCA to identify critical program components that produced desired participant outcomes in SFP 10-14. We have contributed to the understanding of SFP 10-14 specifically, and community-based, family-focused programs in general, by identifying various combinations of factors that lead to program sustainment. Despite an increase in the number of studies focusing on program sustainment, there remains a relative dearth of empirical evidence on the combinations of factors associated with sustainment [15, 16, 59], especially those guided by established sustainment frameworks and measurement tools [23]. In addition to identifying the one necessary condition (environmental support) for SFP 10-14 program sustainment, our study also provides empirical evidence that multiple pathways lead to program sustainment success. These results reinforce the multidimensional and interactive nature of sustainability capacity domains and their role in program sustainment [21, 27, 30]. The current study has improved the understanding of pathways to sustainment by (a) using an established conceptual framework and the standardized PSAT tool to focus on domains identified in the literature as influencing sustainment and (b) using QCA to study causal complexities of sustainment [60].

Although the present study makes important contributions to the study and support of sustainment, these results must be interpreted within the context of several potential limitations. First, the small sample size in the current study might have limited our ability to identify all possible combinations of factors that could lead to program sustainment. Second, the results may be potentially biased due to the QCA requirement of complete data on all variables included in the truth table. Third, we did not use any missing data techniques to account for any potential bias caused by systematic missing data. Fourth, the dichotomization of sustainability domains and outcomes as being present or absent might have obscured the potential influence of domains that contribute in some measure to sustainment and our chosen calibration method (i.e., mean-split) may have limited the generalizability of the findings. We did however complete a series of sensitivity analyses to compare the mean-split calibration to other cutpoints and found highly similar results. Also, it is important to note that the current study relied on a single-reporter to assess both the conditions and sustainment success of the SFP 10-14 program being implemented by their organization. It is also possible (although unlikely given that the database from which our sample was recruited included a single contact person per program delivery) that multiple staff from the same site/organization completed our survey. To the extent that this happened, and to the extent respondents from the same site reported similar perceptions of their organization’s capacity and SFP 10-14 sustainment, this may have artificially inflated associations between these variables. Finally, we did not include the effect of implementer characteristics, as well as other intervention characteristics that research has shown to be associated with sustainability in community-based programs [14, 61].

## Conclusions

In order to achieve wide-scale impact in community settings, programs must be sustained. Theory and empirical evidence suggest that intervention characteristics, organizational context, capacity for program implementation, and processes related to implementation are associated with continued program delivery. However, few studies examine how combinations of these factors work together in different settings to influence program sustainment. The present study helped move us forward in this regard by using an innovative case-oriented technique to uncover multiple pathways to the sustainment of one community-based youth substance misuse prevention program, SFP 10-14. Future studies should aim to extend and determine the generalizability of these findings by using QCA to determine whether similar or different combinations of conditions are necessary and/or sufficient for achieving the sustainment of other types of prevention programs. Leveraging other features of QCA such as fuzzy set (which can accommodate continuous variables) and complementing the quantitative analyses with in-depth qualitative data for each case would also improve our understanding of the multiple pathways to sustainment. We did conduct semi-structured interviews with a subset of the present study’s sample and analyses are currently in progress. These will be presented in a separate paper where we plan to compare results from the quantitative and qualitative data and further explain the patterns uncovered via the QCA analyses. Ultimately, results from this body of work should form the foundation needed to develop and test evidence-based sustainment strategies and support systems that acknowledge and allow for the various needs of diverse programs working in diverse settings.

## Data Availability

The datasets used and/or analyzed during the current study are available from the corresponding author on reasonable request.

## Abbreviations

SFP 10-14: Strengthening Families Program for Parents and Youth 10-14
PSAT: Program Sustainability Assessment Tool
QCA: Qualitative Comparative Analysis
EPIS: Exploration, Preparation, Implementation, and Sustainment
